# Modulation of docetaxel-induced apoptosis and cell cycle arrest by all- *trans* retinoic acid in prostate cancer cells

**DOI:** 10.1054/bjoc.2001.1818

**Published:** 2001-06

**Authors:** A Nehmé, P Varadarajan, G Sellakumar, M Gerhold, H Niedner, Q Zhang, X Lin, R D Christen

**Affiliations:** Department of Medicine and the Cancer Center, University of California, 9500 Gilman Drive, La Jolla, San Diego, CA 92093-0058

**Keywords:** docetaxel, all- *trans* retinoic acid, prostate cancer, apoptosis, cell cycle

## Abstract

We report that all- *trans* retinoic acid (ATRA) enhanced the toxicity of docetaxel against DU145 and LNCaP prostate cancer cells, and that the nature of the interaction between ATRA and docetaxel was highly synergistic. Docetaxel-induced apoptotic cell death was associated with phosphorylation and hence inactivation of Bcl-2. ATRA enhanced docetaxel-induced apoptosis and combined treatment with ATRA and docetaxel resulted in down-regulation of Bcl-2. Docetaxel caused phosphorylation and hence inactivation of cdc2 kinase result ing in G2/M arrest. ATRA inhibited docetaxel-induced phosphorylation of cdc2 resulting in activation of cdc2 kinase and partial reversal of the G2/M arrest. ATRA also inhibited docetaxel-induced activation of MAPK indicating that the effects of docetaxel and ATRA on cdc2 phosphorylation are dependent on MAPK. We conclude that ATRA synergistically enhances docetaxel toxicity by down-regulating Bcl-2 expression and partially reverses the docetaxel-induced G2/M arrest by inhibiting docetaxel-induced cdc2 phosphorylation in a pathway that is dependent on MAPK. © 2001 Cancer Research Campaign http://www.bjcancer.com


*British Journal of Cancer* (2001) **84**(11), 1571–1576
© 2001 Cancer Research Campaign
doi: 10.1054/ bjoc.2001.1818, available online at http://www.idealibrary.com on IDEAL®

### Modulation of docetaxel-induced apoptosis and cell cycle arrest by all-*trans* retinoic acid in prostate cancer cells

**A Nehmé, P Varadarajan, G Sellakumar, M Gerhold, H Niedner, Q Zhang, X Lin and RD Christen**

Department of Medicine and the Cancer Center, University of California, San Diego, 9500 Gilman Drive, La Jolla, CA 92093-0058

**Summary** We report that all-*trans* retinoic acid (ATRA) enhanced the toxicity of docetaxel against DU145 and LNCaP prostate cancer cells, and that the nature of the interaction between ATRA and docetaxel was highly synergistic. Docetaxel-induced apoptotic cell death was associated with phosphorylation and hence inactivation of Bcl-2. ATRA enhanced docetaxel-induced apoptosis and combined treatment with ATRA and docetaxel resulted in down-regulation of Bcl-2. Docetaxel caused phosphorylation and hence inactivation of cdc2 kinase result ing in G2/M arrest. ATRA inhibited docetaxel-induced phosphorylation of cdc2 resulting in activation of cdc2 kinase and partial reversal of the G2/M arrest. ATRA also inhibited docetaxel-induced activation of MAPK indicating that the effects of docetaxel and ATRA on cdc2 phosphorylation are dependent on MAPK. We conclude that ATRA synergistically enhances docetaxel toxicity by down-regulating Bcl-2 expression and partially reverses the docetaxel-induced G2/M arrest by inhibiting docetaxel-induced cdc2 phosphorylation in a pathway that is dependent on MAPK. © 2001 Cancer Research Campaign http://www.bjcancer.com

**Keywords**: docetaxel; all-*trans* retinoic acid; prostate cancer; apoptosis; cell cycle

Prostate carcinoma is the second leading cause of death from cancer among men (Catalona, 1994). Prostate cancer is an androgen-dependent tumour, therefore hormonal therapy is often used as a primary treatment option for men with symptomatic, advanced disease. However, 20% of patients are refractory to treatment (Sinha et al, 1977). Furthermore, virtually all patients who exhibit an initial therapeutic response will relapse within 3 years with androgen-independent carcinoma that is rapidly fatal (Sinha et al, 1977; Catalona, 1994).

Recently, docetaxel has been shown to have marked activity against prostate cancer cells both in vitro and in vivo. Docetaxel has been shown to induce programmed cell death in DU145, LNCaP and PC-3 prostate cancer cells (Haldar et al, 1995, 1996, 1997, 1998). Treatment of prostate cancer cells with docetaxel induces phosphorylation of Bcl-2 and abrogates the normal anti-apoptotic function of Bcl-2 (Haldar et al, 1995,1996,1997,1998).

Petrylak et al have recently shown that the combination of docetaxel and estramustine is active and well tolerated in patients with androgen-independent prostate cancer (Petrylak et al, 1999). The PSA response rate in this phase I trial was 63%. Similarly, a phase II study of docetaxel, estramustine and low-dose hydrocortisone in men with hormone-refractory prostate cancer conducted by the Cancer and Leukemia Group B showed a response rate of 69% (Savarese et al, 1999). Monotherapy with docetaxel has also been shown to be active in patients with androgen-independent prostate cancer with an objective response rate of 46% (Picus and Schultz, 1999).

*Received 7 November 2000*
*Revised 23 February 2001*
*Accepted 26 February 2001*

*Correspondence to:* R Christen

Retinoids include natural as well as synthetic derivatives of vitamin A and have been shown to modulate cellular growth and differentiation of normal and neoplastic epithelial cells by interacting with nuclear receptors that function as retinoid-dependent transcriptional factors, including the RAR and RXR receptors (Amos and Lotan, 1990; Mangelsdorf et al, 1994). All-*trans* retinoic acid (ATRA) induces growth arrest and differentiation of diverse tumour cell lines in vitro (Caliaro et al, 1994; Lotan, 1994). Recent studies have shown that ATRA enhances the cytotoxicity of chemotherapeutic agents. For instance, ATRA has been shown to increase the in vitro sensitivity to cisplatin in squamous head and neck cancer and in ovarian cancer cells (Sacks et al, 1995; Aebi et al, 1997; Caliaro et al, 1997). Formelli et al have demonstrated in an in vivo model that the synthetic retinoid fenretinide enhanced activity of cisplatin and increased survival of nude mice bearing ovarian carcinoma xenografts (Formelli and Cleris, 1993). Encouraging clinical results have also been reported for using retinoids and cytotoxic agents in combination (Seiter et al, 2000).

In the present study, we investigated the effects of ATRA on docetaxel-induced cell death and cell cycle arrest in human prostate cancer cell lines. In addition, we investigated the signalling pathways which are activated in response to treatment with ATRA and docetaxel and which regulate apoptotic cell death and progression through the cell cycle.

### MATERIALS AND METHODS

### Cell culture

The human hormone-independent DU145 and hormone-dependent LNCaP prostate cancer cell lines were obtained from American Type Culture Collection (Rockville, MD, ATCC #HTB-81 and

#CRL-1740 for DU145 and LNCaP cell lines, respectively). DU145 cells and LNCaP cells were cultured in Eagle's MEM (Irvine Scientific, Irvine, CA) and RPMI 1640 (Irvine Scientific), respectively. Medium was supplemented with 100 mM L-glutamine and 10% heat-inactivated fetal bovine serum.

### Reagents

ATRA was purchased from Sigma Chemical Co (St Louis, MO) and was dissolved in DMSO as 50mM stock solution. Docetaxel was obtained from Aventis Pharmaceuticals (Collegeville, PA), and was prepared as a 50 mM stock solution in polysorbate 80.

### Treatment schedule

In order to determine the effects of ATRA and docetaxel on growth inhibition, apoptosis, cell cycle phase distribution, and protein expression, DU145 and/or LNCaP cells were exposed to docetaxel for 1 h and to ATRA continuously starting 72 h prior to docetaxel exposure. The dose of ATRA was fixed at 5 µM. We have previously shown that in head and neck cells, this schedule resulted in maximum enhancement of drug sensitivity (Aebi et al, 1997).

### Growth inhibition assays

Sulforhodamine B growth rate assays were performed according to the protocol described by Monks et al (1991). Control and ATRA treated cells were seeded into 96-well plates at a density of 6000 cells well$^{-1}$ in 100 µl medium. After 24 h, cells were exposed to docetaxel for 1 h and allowed to grow for an additional 72 h thereafter. ATRA-treated cells were exposed to a fixed concentration of 5 µM ATRA continuously starting 72 h prior to docetaxel treatment. Cell growth was stopped by adding 50 µl of 50% (w/v) trichloroacetic acid, and cellular protein was stained with sulforhodamine B and measured by spectrophotometry (Skehan et al, 1990). Control plates were fixed to estimate the cellular protein at time 0. The relative growth rate, *r*, was calculated as reported previously (Skehan et al, 1990). Each experiment was performed in triplicate, and $IC_{50}$ values were estimated by linear interpolation at $r = 0.5$.

### Median effect analysis

Median effect analysis was used to determine the nature of the interaction between docetaxel and ATRA (Chou and Talalay, 1984; Chou and Chou, 1986). The combination index was determined from growth rate assays at increasing levels of growth inhibition (Chou and Talalay, 1984; Chou and Chou, 1986). CI values of less than or greater than one indicate synergy and antagonism, respectively; whereas a CI value of 1 indicated additivity of the drugs. Drugs were combined at a ratio equal to the ratio of the $IC_{50}$ values for each drug determined by growth rate assay. The combination was compared to the cytotoxicity of each drug alone in every experiment.

### Detection of apoptotic cells

Cells were exposed to docetaxel for 1 h at an $IC_{20}$ in either the presence of or the absence of ATRA. 48 and 72 h after docetaxel treatment, cells were collected by trypsinization and resuspended in PBS containing 4 µg ml$^{-1}$ acridine orange and 4 µg ml$^{-1}$ ethidium bromide. Subsequently, cells were assessed for apoptotic morphology by supravital fluorescence microscopy. Cells were scored as apoptotic according to established morphologic criteria (McGahon et al, 1995). Cells with intact cytoplasmic membranes, as reflected by green fluorescent condensed nuclei, were scored as early apoptotic, and cells with red fluorescent condensed nuclei as late apoptotic.

### Cell cycle phase distribution

Approximately 1 to $2 \times 10^6$ cells were exposed to 10 nM docetaxel for 1 h in either the presence of or the absence of ATRA. At 0, 1, 2 and 3 days after docetaxel treatment, cells were harvested by trypsinization, washed twice with ice-cold PBS, and fixed in ice-cold 70% ethanol at a concentration of $10^6$ cells ml$^{-1}$. Cells were counted and $10^6$ cells per sample were centrifuged, resuspended in 300 µl of ice-cold PBS and treated with 0.1 mg ml$^{-1}$ RNase A (Sigma Chemical Co) at 37˚C for 30 min. Propidium iodide (Molecular Probes, Eugene, OR) at a final concentration of 50 µg ml$^{-1}$ was then added to the cell suspensions. After a 30 min incubation on ice, cells were analysed on a FACScan flow cytometer (Becton-Dickinson, San Jose, CA). Multicycle AV Cell Cycle software (Phoenix Flow Systems, San Diego, CA) was used to calculate the fraction of cells in each phase of the cell cycle, as described previously by Dean and Jett (1974).

### Protein extraction and Western blotting

Logarithmically growing cells were treated with docetaxel for 1 h at an $IC_{20}$ in either the presence of or the absence of ATRA. At 12, 24, 48 and 72 h after the beginning of docetaxel exposure, cells were lysed in 100 µl of lysis buffer (10 mM Tris-HCl (pH 7.4), 150 mM NaCl, 5 mM EDTA, 1% Triton X-100, 5 mM DTT, 1 mM sodium vanadate, 0.1 mM phenylmethylsulfonyl fluoride, and 5 mM aminocaproic acid) for 30 min on ice. For the detection of PARP cleavage, DNA was sheared by passing the cell lysates through a 25 gauge needle several times. The insoluble material was removed by centrifugation at 30 000 ***g*** for 20 min at 4˚C. 50 µg of total protein was subjected to electrophoresis on polyacrylamide gels. Total protein was transferred to polyvinylidene difluoride membranes (Immobilon P, Millipore, Bedford, MA) by electroblotting. The blots were stained with the following antibodies: polyclonal anti-phospho-MAPK and anti-phospho-p34$^{cdc2}$ (New England Biolabs, Inc, Beverly, MA), polyclonal anti-Bcl-2 and monoclonal anti-p53 (Santa Cruz Biotechnology), and monoclonal-anti-Bcl-2 (Genosys, Woodland, TX). The monoclonal C-2-10 anti-PARP antibody was from Dr G Poirier (University of Laval, Canada). After washing the blots, horseradish peroxidase-conjugated antirabbit or anti-mouse antibodies diluted 1:3000 (Amersham Life Science Inc, Arlington Heights, IL) were added, and complexes were visualized by enhanced chemiluminescence (Amersham Life Science Inc).

### Densitometry

Levels of protein expression were quantitated using an HP ScanJet 5100 C Scanner (Hewlett-Packard Company, Greeley, CO).

*© 2001 Cancer Research Campaign*

### RESULTS

### Effect of ATRA on docetaxel toxicity

DU145 and LNCaP cells were exposed to increasing concentrations of docetaxel for 1 h in either the presence of or the absence of ATRA (Figure 1). Exposure to ATRA was started 72 h prior to docetaxel and continued for 72 h thereafter. The dose of ATRA was fixed at 5 μM. In LNCaP cells, ATRA caused a 2.5 ± 0.3-fold increase in docetaxel toxicity as quantitated by the ratio of the $IC_{50}$ values ($n$ = 3, $P$ = 0.04 by 2-sided $t$-test). In DU145 cells, ATRA caused a 3.0 ± 0.4-fold increase in docetaxel toxicity ($n$ = 3, $P$ = 0.03 by 2-sided $t$-test).

### Median effect analysis of the interaction between docetaxel and ATRA

The nature of the interaction between docetaxel and ATRA was analysed by the combination index-isobologram method (Chou and Talalay, 1984; Chou and Chou, 1986). This procedure is based on the median effect principle and on isobologram analysis and allows the characterization of drug interactions with a single number, the combination index. Figure 2 shows plots of the combination index as a function of the fraction of cells affected. In both, DU145 and LNCaP cells, the combination index was <1, indicating strong synergy over the first 2 logs of tumour cell kill.

### Effect of ATRA on docetaxel-induced apoptosis

Cells were exposed to docetaxel at an $IC_{20}$ concentration for 1 h in either the presence of or the absence of ATRA. The $IC_{20}$ concentration was 15 nM and 10 nM in DU145 and LNCaP cells, respectively. The fraction of apoptotic cells was determined at 48 and 72 h after docetaxel exposure (Figure 3). In DU145 cells, ATRA enhanced docetaxel-induced apoptosis by 2.2 ± 0.2 fold ($n$ = 3, $P$ = 0.008 by 2-sided $t$-test) and 2.6 ± 0.2-fold ($n$ = 3, $P$ = 0.001), at 48 and 72 h, respectively. In LNCaP cells, ATRA had no significant effect on docetaxel-induced apoptosis at 48 h; however, at 72 h, ATRA enhanced docetaxel-induced apoptosis by 2.2 ± 0.2-fold ($n$ = 3, $P$ = 0.001).

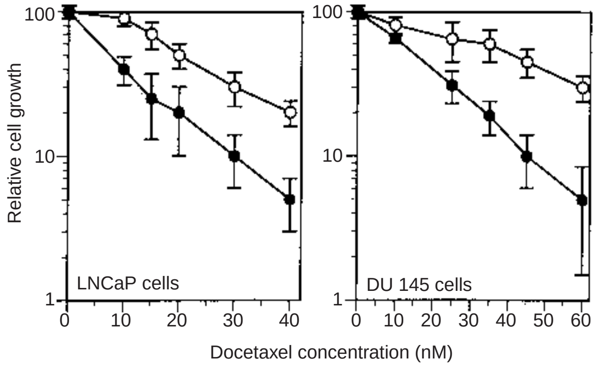

**Figure 1** Effect of ATRA on docetaxel toxicity. LNCaP and DU145 cells were exposed to docetaxel for 1 h in either the presence of or the absence of ATRA. Dose–response curves were determined by sulforhodamine B growth rate assay. Open circles, docetaxel; closed circles, docetaxel plus ATRA. Data points represent mean ± SD of at least 3 independent experiments performed with triplicate cultures

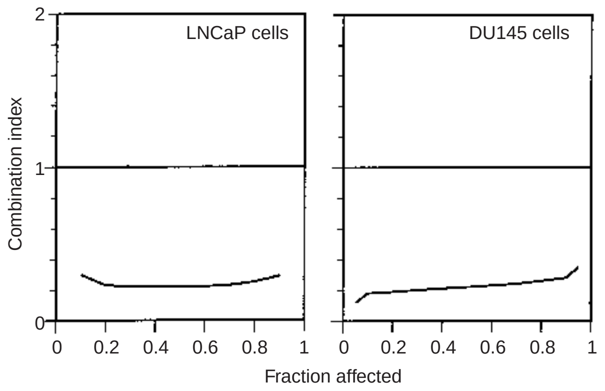

**Figure 2** Combination index plots for the interaction between ATRA and docetaxel in LNCaP and DU145 cells. The curves represent the mean of three independent experiments performed with triplicate cultures

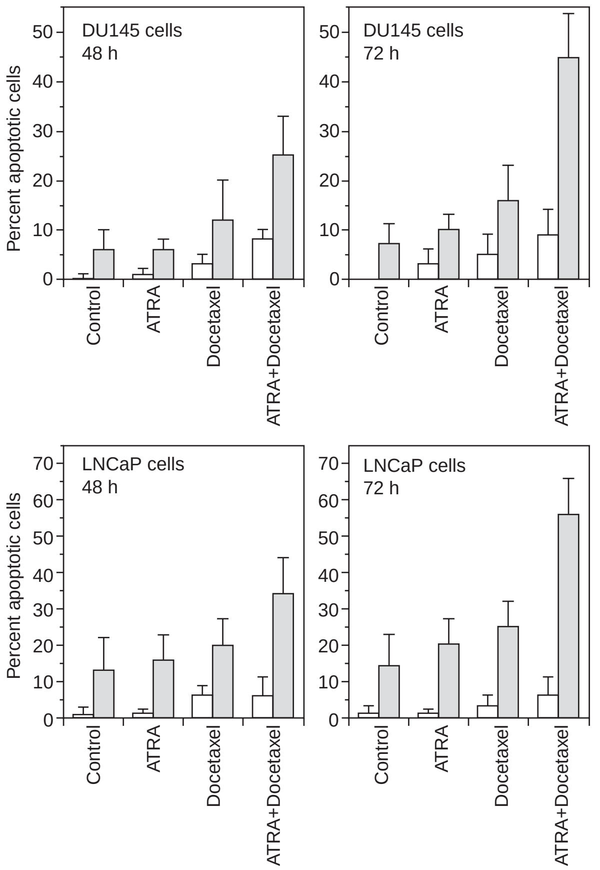

**Figure 3** Effect of ATRA on docetaxel-induced apoptosis. DU145 and LNCaP cells were exposed to docetaxel for 1 h at an $IC_{20}$ in either the presence of or the absence of ATRA. Open columns, early apoptotic cells; shaded columns, late apoptotic cells. Columns represent mean ± SD of 3 independent experiments performed with triplicate cultures

### Effect of ATRA and docetaxel on Bcl-2 expression and phosphorylation

We have previously shown that ATRA enhances the sensitivity of head and neck cancer cells by down-regulating Bcl-2 expression

© 2001 Cancer Research Campaign

(Aebi et al, 1997). In prostate cancer cells, microtubule-damaging agents have been shown to induce phosphorylation of Bcl-2 Haldar et al,1994,1995,1996,1997). Phosphorylation of Bcl-2 can be demonstrated by the appearance of a slow mobility form of Bcl-2 (Haldar et al, 1996; 1997). Bcl-2 phosphorylation has been shown to interfere with the antiapoptotic function of Bcl-2 (Haldar et al, 1995). Since DU145 cells do not express Bcl-2 (Haldar et al, 1996), we determined the effects of docetaxel on Bcl-2 expression and phosphorylation only in LNCaP cells.

LNCaP cells were exposed to 10 nM docetaxel for 1 h ($IC_{20}$) in either the presence of or the absence of ATRA (Figure 4). Docetaxel treatment resulted in the appearance of a slow mobility form of Bcl-2 which corresponds to the phosphorylated form of Bcl-2 (Haldar et al, 1996, 1997). The slow mobility form of Bcl-2 was most prominent at 24 h after exposure to docetaxel. Treatment with ATRA alone had no major effect on Bcl-2 expression and phosphorylation. However, combined treatment with ATRA and docetaxel resulted in down-regulation of Bcl-2 expression which was most marked at 48 h.

### Effect of ATRA and docetaxel on PARP cleavage

To further assess apoptotic cell death, we investigated the effects of ATRA and docetaxel on PARP cleavage. PARP is a specific substrate of the apopain/CPP32 protease (Nicholson et al, 1995). The appearance of an 85 kD fragment indicates cleavage of PARP by apopain/CPP32 (Kaufmann et al, 1993). In both, LNCaP and DU145 cells, treatment with docetaxel resulted in PARP cleavage which was most prominent at 48 h (Figures 4 and 5). Treatment with ATRA alone resulted in the appearance of a faint 85 kD PARP fragment. In LNCaP cells, ATRA markedly enhanced docetaxel-induced PARP cleavage at 24 h.

### Effect of ATRA and docetaxel on p53 expression

In LNCaP and DU145 cells, ATRA and docetaxel had no effect on the level of expression of p53 (Figures 4 and 5).

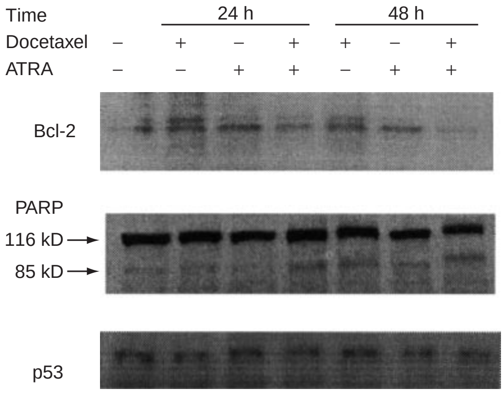

**Figure 4** Effect of ATRA and docetaxel on expression of Bcl-2, PARP and p53 in LNCaP cells. Cells were exposed to 10 nM docetaxel for 1 h ($IC_{20}$) in either the presence of or the absence of ATRA. Cellular proteins were analysed by SDS PAGE and Western blotting. Bcl-2 expression and phosphorylation. Treatment with docetaxel resulted in the appearance of a slow mobility form of Bcl-2 which has been shown to correspond to the phosphorylated form of Bcl-2 (Haldar et al, 1995, 1996). Combined treatment with ATRA and docetaxel resulted in down-regulation of Bcl-2 expression. PARP cleavage. Treatment with docetaxel resulted in cleavage of PARP. ATRA markedly enhanced docetaxel-induced PARP cleavage at 24 h. p53 expression. ATRA and docetaxel had no effect on the expression of p53

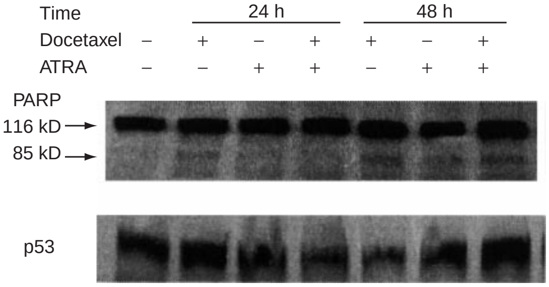

**Figure 5** Effect of ATRA and docetaxel on expression of PARP and p53 in DU145 cells. Cells were exposed to 15 nM docetaxel for 1 h ($IC_{20}$) in either the presence of or the absence of ATRA. In DU145 cells, docetaxel treatment resulted in PARP cleavage at 24 and 48 h. ATRA and docetaxel had no effect on the expression of p53

### Effect of ATRA and docetaxel on cell cycle phase distribution

DU145 cells were exposed to 15 nM docetaxel for 1 h which corresponded to an $IC_{20}$ and $IC_{40}$ in the absence of and in the presence of ATRA, respectively. Treatment with ATRA alone caused a G1 arrest (Figure 6). Treatment with docetaxel alone caused a marked and prolonged G2/M arrest which peaked at 24 and 48 h. Compared with docetaxel treatment, combined treatment with ATRA and docetaxel caused a G2/M arrest that was much less prominent and more rapidly reversible. Similar results were obtained in LNCaP cells (data not shown).

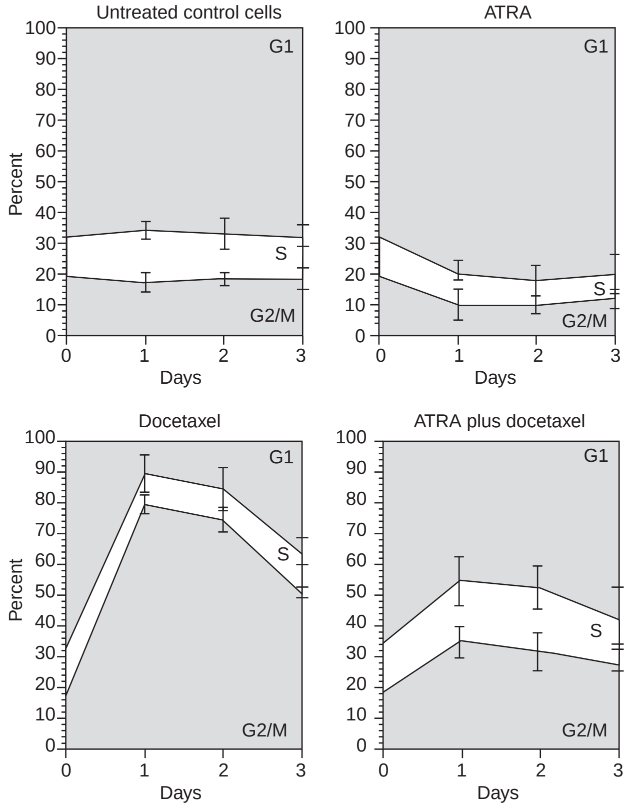

**Figure 6** Effect of ATRA and docetaxel on cell cycle phase distribution. DU145 cells were exposed to 15 nM docetaxel for 1 h ($IC_{20}$) in either the presence of or the absence of ATRA. Cell cycle phase distribution was determined by flow cytometry. Data points represent mean ± SD of 3 different experiments

*© 2001 Cancer Research Campaign*

### Effect of ATRA on docetaxel induced G2/M arrest

In order to better quantitate the effect of ATRA on docetaxel-induced G2/M arrest, we calculated the area under the curve for the fraction of cells in each phase of the cell cycle over time (percent × days). The AUC for the G2/M phase was 193 ± 20 (percent of cells in G2/M phase × days, $n$ = 3, mean ± SD) in docetaxel-treated DU145 cells. Combined treatment with ATRA and docetaxel reduced the AUC for the G2/M phase to 92 ± 15 (percent of cells in G2/M phase × days, $n$ = 3, mean ± SD). This represents a 50% reduction in G2/M arrest which was statistically significant ($n$ = 3, $P$ = 0.002 by 2-sided $t$-test). Similarly, ATRA reduced the AUC for the G2/M phase in docetaxel-treated LNCaP cells by 43 ± 6% ($n$ = 3, mean ± SD, $P$ = 0.006 by 2-sided $t$-test).

### Effect of ATRA and docetaxel on p34 cdc2 phosphorylation cdc2 plays a critical role in the G2/M transition. The phosphorylated form of cdc2 is inactive resulting in G2/M arrest (Shapiro and Hapre, 1999). DU145 cells were exposed to 15 nM docetaxel for 1 h ($IC_{20}$) in either the presence of or the absence of ATRA. Docetaxel treatment resulted in phosphorylation of cdc2 (Figure 7). Phosphorylated cdc2 was most abundant at 48 h after docetaxel treatment. Treatment with ATRA did not have a major effect on cdc2 phosphorylation. However, when combined with docetaxel, ATRA reduced docetaxel-induced cdc2 phosphorylation at 48 h, resulting in activation of cdc2 and partial reversal of docetaxel-induced G2/M arrest.

### Effect of ATRA and docetaxel on activation of MAPK

Microtubule damaging agents have been shown to activate MAPK without altering the level of MAPK expression (Ding et al, 1996; Wang et al, 1998; Shtil et al, 1999). We therefore investigated the effects of ATRA and docetaxel on MAPK activity. MAPK activity was determined by immunoblotting using an antibody directed against the phosphorylated form of MAPK. Phosphorylation of MAPK has been shown to result in MAPK activation (Lin et al, 1995). In DU145 cells, docetaxel treatment resulted in phosphorylation of p44 and p42 MAPK which peaked at 48 h (Figure 7). Treatment with ATRA alone had no major effect on p44/p42 MAPK phosphorylation. However, ATRA markedly reduced docetaxel-induced MAPK phosphorylation.

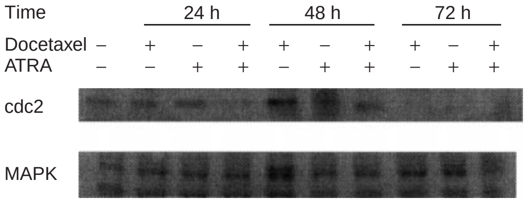

**Figure 7** Effect of ATRA and docetaxel on cdc2 phosphorylation and MAPK activity. DU145 cells were exposed to 15 nM docetaxel for 1 h ($IC_{20}$) in either the presence of or the absence of ATRA. Treatment with docetaxel resulted in phosphorylation of cdc2 which was most prominent at 48 h. ATRA alone had no effect of cdc2 phosphorylation, however ATRA reduced docetaxel-induced cdc2 phosphorylation at 48 h. In addition, ATRA inhibited docetaxel-induced phosphorylation and activation of MAPK

### DISCUSSION

Prostate cancer cells are intrinsically resistant to many chemotherapeutic agents. Therefore, novel strategies aimed at reversing drug resistance are urgently needed. We report that ATRA enhances the in vitro toxicity of docetaxel against prostate cancer cells, and that the nature of the interaction between ATRA and docetaxel is truly synergistic in nature. In addition, we demonstrate that docetaxel induces apoptotic cell death in prostate cancer cells and that ATRA enhances docetaxel-induced apoptosis.

Our results confirm previous reports indicating that treatment of prostate cancer cells with docetaxel results in phosphorylation of Bcl-2 (Haldar et al, 1997). Phosphorylation of Bcl-2 at serine residues has been shown to interfere with the anti-apoptotic function of Bcl-2 by preventing binding of Bcl-2 to the pro-apoptotic protein bax (Haldar et al, 1995). Treatment of LNCaP cells with ATRA alone had no major effect on the expression or phosphorylation of Bcl-2. However, combined treatment with ATRA and docetaxel caused down-regulation of Bcl-2. This observation is in line with previous reports indicating that ATRA combined with cytotoxic agents results in downregulation of Bcl-2 expression and enhanced drug sensitivity (Hu et al, 1995, 1998; Bradbury et al, 1996; Nagy et al, 1996; Aebi et al, 1997; Pisani et al, 1997; Andreeff et al, 1999).

Docetaxel-induced microtubule damage caused a marked and prolonged G2/M arrest which peaked at 24 and 48 h and which was partially reversed by ATRA. The G2/M arrest coincided with docetaxel-induced phosphorylation and inactivation of cdc2. In the G2/M transition, the cyclin-dependent protein kinase complex, cdc2 cyclin B1 complex, plays a critical role (Shapiro and Hapre, 1999). The kinase activity of cdc2 is controlled during the cell cycle both by its association with cyclin B1 and by phosphorylation and dephosphorylation on the inhibitory phosphorylation sites. The phosphorylated form of cdc2 is inactive resulting in G2/M arrest.

In DU145 cells, treatment with docetaxel resulted in phosphorylation of MAPK which also peaked at 48 h. Phosphorylation of MAPK has been shown to be associated with activation of MAP kinase activity (Lin et al, 1995), and activation of MAPK has been shown to result in phosphorylation of cdc2 resulting in inactivation of cdc2 and G2/M arrest (Bitangcol et al, 1998). Thus our findings suggests that docetaxel-induced microtubule damage triggers a signalling pathway that involves activation of MAPK resulting in phosphorylation and hence inactivation of cdc2 which, in turn, results in G2/M arrest.

Treatment of DU145 cells with ATRA alone had no major effect on the phosphorylation status of MAPK and cdc2. However, ATRA inhibited docetaxel-induced phosphorylation of MAPK and cdc2. Inhibition of docetaxel-induced phosphorylation of cdc2 resulted in activation of cdc2 and partial reversal of the docetaxel-induced G2/M arrest.

We conclude that ATRA synergistically enhances docetaxel toxicity by down-regulating Bcl-2 expression and that ATRA partially reverses the docetaxel-induced G2/M arrest by inhibiting docetaxel-induced cdc2 phosphorylation in a pathway that is dependent on MAPK.

### ACKNOWLEDGEMENTS

This work was supported by grant 10044 from Aventis Pharmaceuticals, grant NI602/1 from the German Research Foundation (DFG) and by grants from the Colleen Gilbert Foundation, Moves

*© 2001 Cancer Research Campaign* 

Fitness, CaP CURE, and the Clayton Foundation for Research – California Division. Xinjian Lin and Randolph Christen are Clayton Foundation Investigators.

### REFERENCES

Aebi S, Kroning R, Cenni B, Sharma A, Fink D, Los G, Weisman R, Howell SB and Christen RD (1997) all-trans retinoic acid enhances cisplatin-induced apoptosis in human ovarian adenocarcinoma and in squarmous head and neck cancer cells. *Clin Cancer Res* **3**: 2033–2038

Amos B and Lotan R (1990) Retinoid-sensitive cells and cell lines. *Methods Enzymol* **190**: 217–225

Andreeff M, Jiang S, Zhang Z, Konopleva M, Estrov Z, Snell VE, Xie Z, Okcu MF, Sanchez-Williams G and Dong J (1999) Expression of Bcl-2-related genes in normal and AML progenitors: changes induced by chemotherapy and retinoic acid. *Leukemia* **13**: 1881–1892

Bitangcol JC, Chau AS, Stadnick E, Lohka MJ, Sicken B and Shibuya EK (1998) Activation of the p42 mitogen-activated protein kinase pathway inhibits cdc2 activation and entry into M-phase in cycling Xenopus egg extracts. *Molecular Biology of the Cell* **9**: 451–467

Bradbury DA, Aldington S, Zhu YM and Russell NH (1996) Down-regulation of bcl-2 in AML blasts by all-trans retinoic acid and its relationship to CD34 antigen expression. *British J Hematol* **94**: 671–675

Caliaro MJ, Marmouget C, Guichard S, Mazars P, Valette A, Moisand A, Bugat R and Jozan S (1994) Response of four human ovarian carcinoma cell lines to all-trans retinoic acid: relationship with induction of differentiation and retinoic acid receptor expression. *Int J Cancer* **56**: 743–748

Caliaro MJ, Vitaux P, Lafon C, Lochon I, Nehmé A, Valette A, Canal P, Bugat R and Jozan S (1997) Multifactorial mechanism for the potentiation of cisplatin (CDDP) cytotoxicity by all-trans retinoic acid (ATRA) in human ovarian carcinoma cell lines. *Br J Cancer* **75**: 333–340

Catalona WJ (1994) Management of cancer of the prostate. *The New England Journal of Medicine* **331**: 996–1004

Chou J and Chou TC (1986) Multiple drug-effect analysis. In: *Dose-effect analysis with microcomputers*, Chou J and Chou TC (eds) pp 19–64. Elsevier Science Publishing Company, Inc: Amsterdam, Netherlands

Chou TC and Talalay P (1984) Quantitative analysis of dose-effect relationships: the combined effects of multiple drugs or enzyme inhibitors. *Adv Enz Regulation* **22**: 27–55

Dean PN and Jett JH (1974) Mathematical analysis of DNA distributions derived from flow microfluorometry. *J Cell Biol* **60**: 523–527

Ding A, Chen B, Fuortes M and Blum E (1996) Association of mitogen-activated protein kinases with microtubules in mouse macrophages. *J Exp Med* **183**: 1899–1904

Formelli F and Cleris L (1993) Synthetic retinoid fenretinide is effective against a human ovarian carcinoma xenograft and potentiates cisplatin activity. *Cancer Res* **53**: 5374–5376

Haldar S, Negrini M, Monne M, Sabbioni S and Croce CM (1994) Down-regulation of *bcl-2* by *p53* in breast cancer cells. *Cancer Res* **54**: 2095–2097

Haldar S, Jena N and Croce CM (1995) Inactivation of Bcl-2 by phosphorylation. *Proc Natl Acad Sci USA* **92**: 4507–4511

Haldar S, Chintapalli J and Croce CM (1996) Taxol induces *bcl-2* phosphorylation and death of prostate cancer cells. *Cancer Res* **56**: 1253–1255

Haldar S, Basu A and Croce CM (1997) Bcl2 is the guardian of microtubule integrity. *Cancer Res* **57**: 229–233

Haldar S, Basu A and Croce CM (1998) Serine-70 is one of the critical sites for drug-induced Bcl-2 phosphorylation in cancer cells. *Cancer Res* **58**: 1609–1615

Hu ZB, Minden MD and McCulloch EA (1995) Direct evidence for the participation of bcl-2 in the regulation by retinoic acid of the Ara-C sensitivity of leukemic stem cells. *Leukemia (Baltimore)* **9**: 1667–1673

Hu ZB, Minden MD and McCulloch EA (1998) Phosphorylation of BCL-2 after exposure of human leukemic cells to retinoic acid. *Blood* **92**: 1768–1775

Kaufmann SH, Desnoyers S, Ottaviano Y, Davidson NE and Poirier GG (1993) Specific proteolytic cleavage of poly(ADP-ribose)polymerase: an early marker of chemotherapy-induced apoptosis. *Cancer Res* **53**: 3976–3985

Lin A, Minden A, Martinetto H, Claret FX, Lange Carter C, Mercurio F, Johnson GL and Karin M (1995) Identification of a dual specificity kinase that activates the Jun kinases and p38-Mpk2. *Science* **268**: 286–290

Lotan R (1994) Suppression of squamous cell carcinoma growth and differentiation by retinoids. *Cancer Res* (Suppl.) **54**: 1987s–1990s

Mangelsdorf DJ, Umesono K and Evans RM (1994) The retinoid receptors. In: Sporn MB, Roberts AB and Goodman DS (eds), *The Retinoids Biology Chemistry and Medicine Ed. 2*, pp. 319–349 New York: Raven Press

McGahon AJ, Martin SM, Sissonnette RP, Mahboubi A, Shi Y, Mogil RJ, Nishioka WK and Green DR (1995) The end of the cell line: Methods for the study of apoptosis. In *Cell Death*, Schwarz LM and Osborne BA (eds), Vol. 46. Academic Press: San Diego

Monks A, Scudiero D, Skehan P, Shoemaker R, Paull K, Vistica D, Hose C, Langley J, Cronise P, Vaigro-Wolff A, Gray-Woodrich M, Campbell H, Mayo J and Boyd M (1991) Feasibility of a high-flux anticancer drug screen using a diverse panel of cultured human tumor cell lines. *J Natl Cancer Inst* **83**: 757–766

Nagy L, Thomazy VA, Chandraratna RAS, Heyman RA and PJA D (1996) Retinoid-regulated expression of bcl-2 and tissue transglutaminase during the differentiation and apoptosis of human myeloid leukemia (HL-60) cells. *Leukemia Res* **20**: 499–505

Nicholson DW, Ali A, Thomberry NA, Vaillancourt JP, Ding CK, Gallant M, Gareau Y, Griffin PR, Labelle M, Lazebnik YA, Munday NA, Raju SM, Smulson ME, Yamin TT, Yu VL and Miller DK (1995) Identification and inhibition of the ICE/CED-3 protease necessary for mammalian apoptosis. *Nature (Lond.)* **376**: 37–43

Petrylak DP, Macarthur RB, O'Connor J, Shelton G, Judge T, Balog J, Pfaff C, Bagiella E, Heitjan D, Fine R, Zuech N, Swczuk I, Benson M and Olsson CA (1999) Phase I trial of docetaxel estramustine in androgen-independent prostate cancer. *J Clin Oncol* **17**: 858–867

Picus J and Schultz M (1999) Docetaxel as monotherapy in the treatment of hormone-reractory prostate cancer: preliminary results. *Sem Oncol* **26**: 14–18

Pisani F, Del Poeta G, Aronica G, Venditti A, Caravita T and Amadori S (1997) In vitro down-regulation of bcl-2 expression by all-trans retinoic acid in AML blasts. *Ann Hematol* **75**: 145–147

Sacks PG, Harris D and Chou T-C (1995) Modulation of growth and proliferation in squamous cell carcinoma by retinoic acid: a rationale for combination therapy with chemotherapeutic agents. *Int J Cancer* **61**: 409–415

Savarese D, Taplin ME, Halabi S, Hars V, Kreis W and Vogelzang N (1999) A phase II study of docetaxel, estramustine and low dose hydrocortisone in men with hormone-refractory prostate cancer: preliminary results of cancer and leukemia group B Trial 9780. *Sem Oncol* **26**: 39–44

Seiter K, Feldman EJ, H, D, Deptala A, Traganos F, Burke HB, Hoang A, Goff H, Pozzuoli M and Kancherla R (2000) Clinical and laboratory evaluation of all-trans retinoic acid modulation of chemotherapy in patients with acute myelogenous leukemia. *British J Hematol* **108**: 40–47

Shapiro GI and Hapre JW (1999) Anticancer drug targets: cell cycle and checkpoint control. *J Clin Invest* **104**: 1645–1663

Shtil AA, Mandlekar S, Yu R, Walter RJ, Hagen K, Tan TH, Roninson IB and Kong AT (1999) Differential regulation of mitogen-activated protein kinases by microtubule-binding agents in human breast cancer cells. *Oncogene* **18**: 334–384

Sinha AA, Blackard CE and Seal US (1977) A critical analysis of tumor morphology and hormone treatments in the untreated and estrogen-treated responsive and refractory human prostatic carcinoma. *Cancer (Phila)* **40**: 2836–2850

Skehan P, Storeng R, Scudiero D, Monks A, McMahon J, Vistica D, Warren JT, Bokesch H, Kenney S and Boyd MR (1990) New colorimetric cytotoxicity assay for anticancer-drug screnning. *J Natl Cancer Inst* **82**: 1107–1112

Wang TH, Wang HS, Ichijo H, Giannakakou P, Foster JS, Fojo T and Wimalasena J (1998) Microtubule-interfering agents activate c-Jun N-terminal kinase/stress-activated protein kinase through both ras and apoptosis signal-regulating kinase pathways. *J Biol Chem* **273**: 4928–4936

 *© 2001 Cancer Research Campaign*
